# Conditions for the generation of cytotoxic CD4^+^ Th cells that enhance CD8^+^ CTL-mediated tumor regression

**DOI:** 10.1038/cti.2016.46

**Published:** 2016-08-12

**Authors:** Kunyu Li, Margaret Baird, Jianping Yang, Chris Jackson, Franca Ronchese, Sarah Young

**Affiliations:** 1 Department of Pathology, Dunedin School of Medicine, University of Otago, Dunedin, New Zealand; 2 Malaghan Institute of Research, Wellington, New Zealand; 3 Departmemt of Medicine, Dunedin School of Medicine, University of Otago, Dunedin, New Zealand

## Abstract

Adoptive cell therapies (ACTs) using tumor-reactive T cells have shown clinical benefit and potential for cancer treatment. While the majority of the current ACT are focused on using CD8^+^ cytotoxic T lymphocytes (CTL), others have shown that the presence of tumor-reactive CD4^+^ T helper (Th) cells can greatly enhance the anti-tumor activity of CD8^+^ CTL. However, difficulties in obtaining adequate numbers of CD4^+^ Th cells through *in vitro* expansion can limit the application of CD4 Th cells in ACT. This study aims to optimize the culture conditions for mouse CD4 T cells to provide basic information for animal studies of ACT using CD4 T cells. Taking advantage of the antigen-specificity of CD4^+^ Th cells from OT-II transgenic mice, we examined different methodologies for generating antigen-specific CD4^+^ Th1 cells *in vitro*. We found that cells grown in complete advanced-DMEM/F12 medium supplemented with low-dose IL-2 and IL-7 induced substantial cell expansion. These Th cells were Th1-like, as they expressed multiple Th1-cytokines and exhibited antigen-specific cytotoxicity. In addition co-transfer of these CD4^+^ Th1-like cells with CD8^+^ CTL significantly enhanced tumor regression, leading to complete cure in 80% of mice bearing established B16-OVA. These observations indicate that the CD4^+^ Th1-like cells generated using the method we optimized are functionally active to eliminate their target cells, and can also assist CD8^+^ CTL to enhance tumor regression. The findings of this study provide valuable data for further research into *in vitro* expansion of CD4^+^ Th1-like cells, with potential applications to cancer treatment involving ACT.

Auto-reactive T cells and cellular immune responses against established malignancy have been identified in both human and animal cancer studies.^1, 2^ The anti-tumor responses of autologous T cells can be improved by *ex vivo* manipulation of these cells followed by clonal expansion to a large number in tissue culture. This strategy circumvents the downregulation of T-cell activation and proliferation in the immunosuppressive tumor microenvironment. Cancer treatment with these *ex vivo* reconstituted T cells is termed adoptive cell therapy (ACT). ACT with tumor infiltrating lymphocytes in patients with metastatic melanoma has demonstrated durable objective responses, especially when a prior lymphodepletion regimen was used.^3, 4^ A drawback of these ACT studies was the requirement of systemic IL-2 administration, which induced significant side-effects such as capillary leakage.^5^

The majority of ACT studies focus on evoking CD8^+^ cytotoxic T lymphocytes (CTL) -mediated anti-tumor responses, due to the ability of CD8^+^ CTL to kill tumor cells directly in a major histocompatibilty complex I (MHC-I) -restricted manner. New findings from both animal and clinical studies have highlighted the importance of CD4^+^ Th1 cells in enhancing CD8^+^ CTL response, memory development and overall anti-tumor immunity.^6, 7^ It has also been reported by several groups that both human and murine CD4^+^ Th cells are capable of acquiring a cytotoxic phenotype and function.^8, 9, 10^ A dendritic cell (DC)-based cancer vaccine study in a mouse model of hepatocellular carcinoma has shown that the vaccine-induced anti-tumor response was mediated by CD4^+^ Th cells but not CD8^+^ CTL.^11^ Another vaccine study has also shown that the efficacy of a cancer vaccine was compromised when CD4^+^ Th cells were depleted before tumor challenge.^12^ These findings suggest the importance of CD4^+^ Th cells in the generation of an effective anti-tumor immunity.

To utilize CD4^+^ Th cells in ACT, these cells need to be expanded in tissue culture. Emerging findings from both animal and human studies indicate that intrinsic factors related to the differentiation stage, phenotype and functional characteristics of the adoptively transferred T cells, are crucial for the success of ACT.^13^ *In vitro* expansion of CD8^+^ CTL has been well studied. However, the methodology for antigen-specific CD4^+^ Th cell expansion has yet to be defined for murine cells. Unlike CD8^+^ CTL which can undergo extensive proliferation upon T-cell receptor (TCR) stimulation, CD4^+^ Th cells have been shown to display a restricted proliferative pattern and exhibit proliferative arrest in early divisions.^14^ By using CD4^+^ Th cells from OT-II transgenic mice, we examined the effects of several common γ-chain cytokines, the strength of antigenic stimulation, and tissue culture media on the magnitude of CD4^+^ Th1 cell expansion. We aimed to achieve high-level cell expansion, while generating multi-functional Th1 cells. The functional activity of these *in vitro* expanded cells was evaluated in both an *in vivo* cytotoxic assay and ACT in a mouse model of melanoma.

## Results

### IL-2 and IL-7 induce similar expansion of CD4^+^ Th cell in a dose-dependent manner.

**Figure 1:**
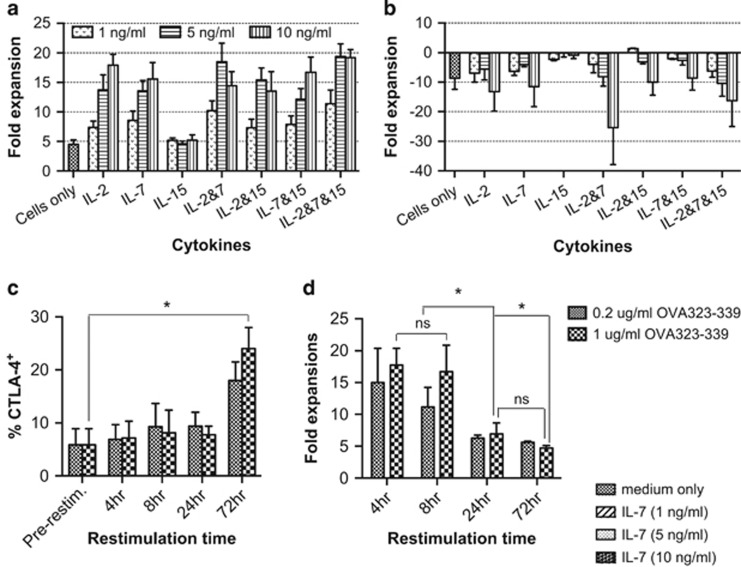
IL-2 and IL-7 induce similar expansion of naive CD4^+^ Th cells, but prolonged antigenic restimulation impairs the secondary cell expansion. CD4^+^ T cells were sorted from splenocytes of naive OT-II mice, and co-cultured with DC-OVA_323-339_ at a DC:T ratio of 1:10 with or without different concentrations of IL-2, IL-7 and IL-15. On day 10, the T cells were restimulated with DC-OVA_323-339_ for secondary expansion until day 20; primary (**a**) and secondary (**b**) expansion. Day 10 CD4^+^ Th cells expanded with IL-2 (1 ng ml^−1^) and IL-7 (5 ng ml^−1^) were restimulated with DC-OVA_323-339_ for different periods of time. Non-adherent T cells were then removed into new wells for expansion with IL-2 (1 ng ml^−1^) and IL-7 (5 ng ml^−1^) until day 20. Surface expression of CTLA-4 on CD4^+^ cells, restimulated for different periods of time, was measured by flow cytometric analysis (**c**). Secondary expansion (**d**). The results are the mean±s.e.m. of three independent experiments. Statistical analysis was done with one-way analysis of variance. *P<0.05.

Cytokines are known to be important to support the survival and proliferation of T cells *in vivo*. To optimize the culture conditions for CD4 T-cell expansion in an antigen-specific manner, we first examined the effect of several major common γ-chain cytokines on cell expansion. IL-2, IL-7 and IL-15 have been shown to be important for T-cell survival and memory development, and are routinely used for CD8^+^ CTL expansion *in vitro*.^15, 16^ We titrated these cytokines into cultures of CD4^+^ Th cells primed with OVA_323-339_-pulsed bone marrow-derived dendritic cells (BMDC) to achieve desirable cell expansion *in vitro* (Supplementary Data Schema S1). The CD4^+^ Th cells were found to expand for only 5 days in the absence of exogenous cytokines (data not shown). Because of this limited expansion, we determined exogenous cytokines were required for the entire cell expansion. IL-2 and IL-7 were found to induce comparable CD4^+^ Th cell expansion in a dose-dependent manner, but did not have a synergistic effect on cell expansion when provided in combination (Figure 1a). IL-15 did not enhance the expansion compared with cells cultured in medium only (Figure 1a). The number of viable T cells was found to slowly decline after 10 days post-TCR stimulation, regardless of the cytokine supplementation (data not shown). Therefore CD4^+^ Th cells were restimulated on day 10 to induce secondary cell expansion. However, massive cell death was observed at 24 h after co-culture of antigen-experienced CD4^+^ Th cell with OVA_323–339_-pulsed BMDC, regardless of the cytokine conditions was used (Figure 1b). This event was not expected, as it has not been reported in the literature.

### A shorter period of restimulation restores antigen-specific cell expansion

To rescue cell death upon restimulation, we examined the association between the duration of TCR stimulation and cell expansion. TCR stimulation can lead to upregulation of CTLA-4 expression, which has been shown to mediate antigen-specific apoptosis of human T cells.^17^ We observed that the expression of CTLA-4 was significantly increased when antigen-experienced cells were co-cultured with OVA_323–339_-pulsed BMDC for 72 h (Figure 1c). However, a significant reduction in T-cell expansion was apparent after only 24 h of restimulation (Figure 1d). Surface expression of CTLA-4 requires intracellular trafficking and endocytosis of this protein, which appears to be continuous following T-cell activation.^18^ This suggests that the program for upregulation of CTLA-4 had been in place before the observation of their surface expression. As such, a short TCR restimulation was used in the further studies to optimize the CD4^+^ Th cell expansion. Survival of these cells in the absence of exogenous cytokines was also examined by resting cells in cytokine free medium after 10 days' clonal expansion. IL-2 and IL-7 induced comparable expansion of antigen-experienced CD4^+^ Th cells after 4 h restimulation (Supplementary Figures S1a-d). However, cells expanded with 5 ng ml^−1^ or higher concentration of IL-2 were found to have poor survival during resting period (Supplementary Figure S1e), suggesting exogenous cytokine-dependent survival of these cells. We also examined IL-7R expression by these cells. No significant difference in IL-7R expression by day 10 cells was observed; however, its expression on day 20 cells was markedly downregulated when the cells were expanded with a cytokine concentration of 10 ng ml^−1^ (Supplementary Figure S2). Therefore IL-7 was used as the main cytokine for CD4^+^ T-cell expansion in further studies. However as IL-2 is involved in the induction of IL-12Rβ2 during Th1 differentiation,^19^ 1 ng ml^−1^ concentration of this cytokine was added only upon antigenic stimulation to initiate Th1 cell differentiation.

### CD4^+^ Th cells expanded in advanced-DMEM/F12 exhibit enhanced secondary expansion

**Figure 2:**
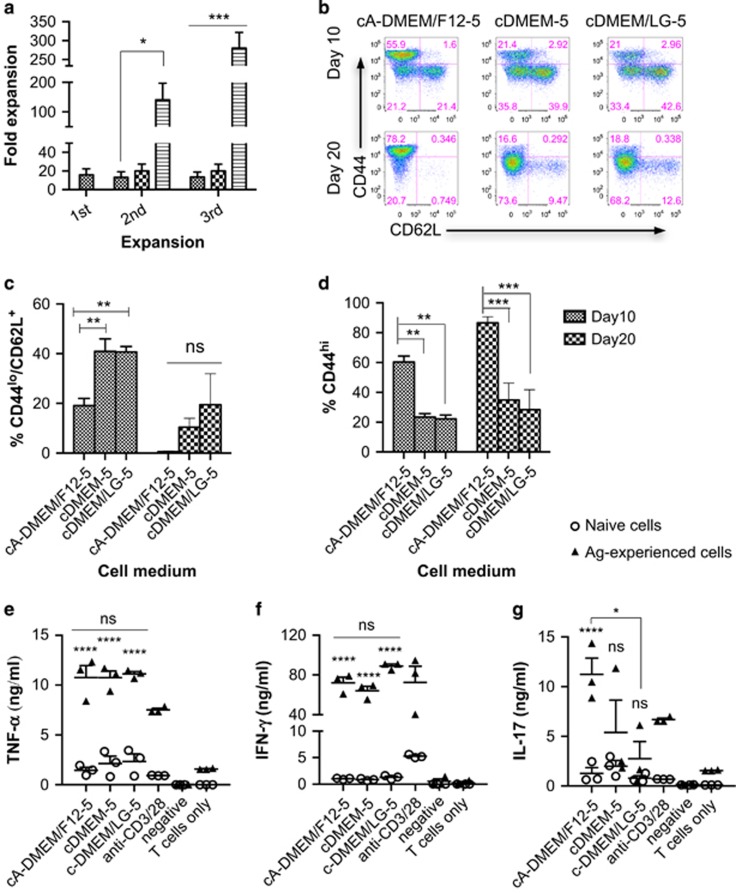
Enhancement of CD4^+^ Th cell expansion in cA-DMEM/F12-5 medium. Naive CD4^+^ OT-II cells were stimulated with DC-OVA_323-339_ and expanded with IL-2 and IL-7 in cDMEM-5 initially. Antigen-experienced cells were restimulated with DC-OVA_323-339_ on days 10 and 20 and expanded in cDMEM-5, cDMEM/LG-5 or c-A-DMEM/F12-5, (**a**) fold expansion of cells. Naive CD4^+^ OT-II cells were stimulated with DC-OVA_323-339_ and expanded with IL-2 and IL-7 in the three different media for 20 days, during which the cells were restimulated with DC-OVA_323-339_ for 4 h on day 10. Expression of CD44 and CD62L after the primary and secondary expansion was examined by flow cytometric analysis (**b**), % CD44^hi^ cells (**c**), % CD62L^+^ cells (**d**). Cell supernatant was collected 3 days after antigenic stimulation for ELISA analysis of IL-17 (**e**), TNF-α (**f**) and IFN-γ (**g**) levels in the supernatant. The results are the mean±s.e.m. of three independent experiments. Statistical analysis was performed with one-way analysis of variance with Bonferroni's multiple comparison test. *P<0.05, **P<0.01, ***P<0.001 and ****P<0.0001.

Previously, we observed a reduction in the expansion capacity of antigen-experienced cells upon TCR restimulation. This suggests generation of late effector cells. Therefore, we were interested to see whether cell expanded in different media could have different secondary expansion capacities. Three different types of media were tested: cDMEM-5, cDMEM/LG-5 and cA-DMEM/F12-5. Complete-DMEM-5 is a basal medium widely used for supporting the growth of many different types of mammalian cells; cDMEM/LG-5 contains three times less glucose compared with cDMEM-5 medium. The advanced medium cA-DMEM/F12-5 is specially formulated with the addition of proteins and trace elements such as ethanolamine, glutathione, ascorbic acid, insulin, transferrin and lipid-rich bovine serum albumin, to support the growth of cells in reduced serum supplementation. Naive CD4^+^ Th cells were initially primed for 10 days, then differentially expanded in the three media after two re-stimulations with DC-OVA_323–339_ (Supplementary Data Schema S2a). Upon restimulation, antigen-experienced Th cells were found to have a similar expansion of 20–30 fold in both cDMEM-5 and cDMEM/LG-5 (Figure 2a). By contrast cells expanded in cA-DMEM/F12-5 had over 100-fold expansion upon the first restimulation (Figure 2a). Because of the massive cell expansion over an extended period of time in cA-DMEM/F12, we hypothesized that these cells might be exhausted. Surprisingly, an even higher cell expansion of over 250-fold was observed upon the second restimulation (Figure 2a) These observations suggest cA-DMEM/F12-5 is preferential for CD4^+^ Th cells without driving cell exhaustion. These three media are also formulated with different glucose contents; and glucose metabolism has been found to affect proliferation, function and memory development in T cells.^20^ We therefore examined the expression of two common memory markers of CD44 and CD62L on and cytokine production by CD4^+^ Th cells after clonal expansion in the three different media (Supplementary Data Schema S2b). No significant difference in CD62L expression was observed (Figures 2b and c). However, cells expanded in cA-DMEM/F12-5 were found to express a significantly higher level of CD44 (Figures 2b and d). Secretion of IFN-γ and TNF-α was similar among CD4^+^ Th cells expanded in the three different media (Figures 2e and f). However, antigen-experienced CD4^+^ Th cells expanded in the cA-DMEM/F12-5 were more capable of IL-17 production (Figure 2g), which has been reported to promote Th1 immunity through upregulating IFN-γ.^21^ As a result of these, cA-DMEM/F12-5 was used for T-cell expansion in the subsequent experiments.

### Induction of Th1-polarized cell expansion by manipulating the levels of IL-12 and signaling through IL-4

**Figure 3:**
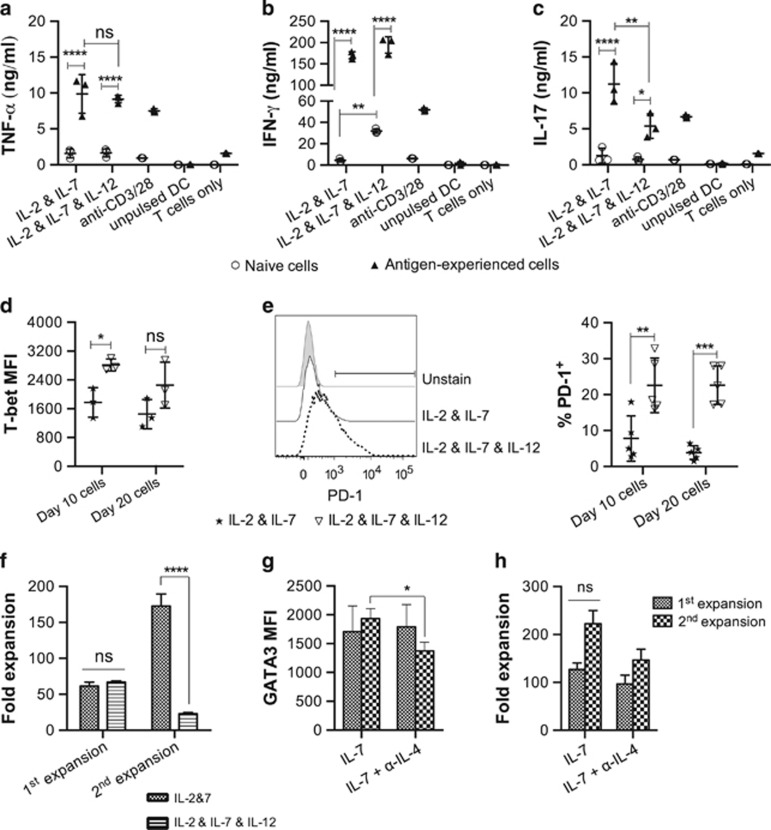
IL-12 expanded cells expressed stronger Th1 phenotypes, but fail to expand following restimulation. Naive CD4^+^ OT-II cells stimulated with DC-OVA_323-339_ and expanded with 1 ng ml^−1^ IL-2 and 5 ng ml^−1^ IL-7 in cA-DMEM/F12 for 20 days, during which, the cells were restimulated on day 10. Either 1 ng ml^−1^ IL-12 or 1 μg ml^−1^ of anti-IL-4 was added in addition to IL-2 and IL-7 to induce Th1 polarization. Cell supernatant was collected 3 days after antigenic stimulation; the amount of TNF-α (**a**), IL-17 (**b**) and IFN-γ (**c**) in the supernatant was measured by ELISA. T-bet expression (**d**), PD-1 expression (**e**) and cell expansion (**f**) with or without the presence of exogenous IL-12, and GATA-3 expression (**g**) and cell expansion (**h**) with or without the presence of anti-IL-4 monoclonal antibodies, were measured. The results are the mean±s.e.m. of three independent experiments. Statistical analysis was done by either Pair *t-*test or one-way analysis of variance with Bonferroni's multiple comparison test. *P<0.05, **P<0.01, ***P<0.0001, ****P<0.0001.

Different subsets of Th cells express a unique set of transcriptional factors.^22^ To identify lineage commitment of CD4^+^ Th cells expanded with IL-2 and IL-7 in cA-DMEM/F12-5, we examined the expression of T-cell transcriptional factors by flow cytometric analysis. Interestingly, these CD4^+^ Th cells were found to express multiple T-cell transcriptional factors including T-bet, GATA-3 and Foxp3 (data not shown) after clonal expansion *in vitro*, suggesting unpolarized Th cell differentiation. IL-12 is known to induce the of differentiation Th1 cell from naive Th cells.^23^ To induce Th1 polarization, 1 ng ml^−1^ IL-12 was added to the cell expansion culture in addition to IL-2 and IL-7 (Supplementary Data Schema S3). This led to an increase of IL-2 and IL-12 secretion with a concomitant decrease in IL-4 and IL-10 secretion in the T cell and DC co-culture (Supplementary Figure S3a). TNF-α secretion was not affected by supplementation of exogenous IL-12 (Figure 3a); however, a significant increase in IFN-γ secretion by naive cells (Figure 3b), and decrease in IL-17 secretion by antigen-experienced cells (Figure 3c), was observed. The increased IFN-γ production by these cells was associated with a trend towards higher T-bet expression (Figure 3d). However, there was no change in GATA-3 and Foxp3 expression (Supplementary Figure S3b). In addition, these cells were found to have an increased level of PD-1 expression (Figure 3e) and a concomitant impairment in secondary expansion (Figure 3f). These observations suggest that prolonged signaling through IL-12 induces a strong effector phenotype with terminal differentiation of these effector Th cells. Therefore to avoid generation of a terminally differentiated phenotype which has been associated with poor cell survival after adoptive transfer,^24^ IL-12 was excluded from subsequent experiments. Diminished IL-4 signaling has also been shown to enhance the Th1 response under suboptimal polarizing conditions.^25^ We therefore utilized anti-IL-4 monoclonal antibodies to induce Th1-polarization (Supplementary Data Schema S3). However, co-expression of T-bet, Foxp3, GATA-3 was again observed regardless of the presence of anti-IL-4 monoclonal antibodies (Figure 3g and Supplementary Figure S4). Although GATA-3 expression was decreased in cells expanded with the addition of anti-IL-4 for 20 days (Figure 3g), no significant difference in cytokine expression was observed (data not shown).

### *In vitro* expanded CD4^+^ Th cells predominantly produce multiple Th1 cytokines, and are cytotoxic, inducing specific lysis of target cells

**Figure 4:**
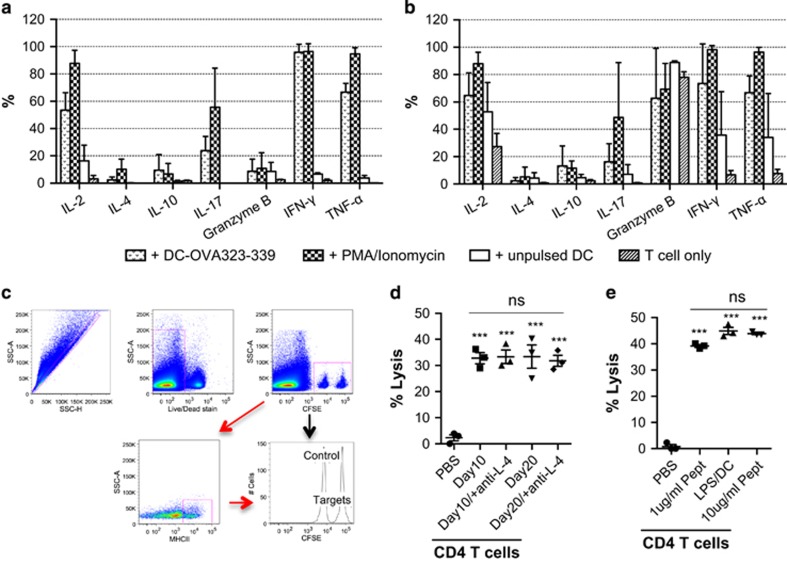
*In vitro* expanded CD4^+^ Th cells predominantly express Th1-cytokines, and exhibit antigen-specific cytotoxicity *in vivo*. Naive CD4^+^ OT-II cells were stimulated with DC-OVA_323-339,_ and expanded with IL-2 and IL-7 in A-DMEM/F12. The cells were restimulated with unpulsed DC, DC-OVA_323-339_ or PMA/Ionomycin in the presence of Brefeldin A, or left untreated for 5 h at 37 °C/5% CO_2_ on day 10 (**a**) and day 20 (**b**). Intracellular expression of T-cell cytokines was measured by intracellular staining and analyzed by flow cytometry. Naive C57BL/6-recipient mice received an i.v. injection of PBS or *in vitro* expanded CD4^+^ Th cells. Donor splenocytes consisted of equal numbers of OVA_323-339_-pulsed/CFSE^Hi^ and unpulsed /CFSE^Lo^ cells and were i.v. injected into all recipient mice 24 h post T-cell transfer. The mice were killed 40 h post-target cell injection, and splenocytes were isolated for flow cytometric analysis of specific lysis of the target cell population (**c**). Lysis of MHC-II^+^ cells by CD4^+^ Th cells expanded with or without anti-IL-4 (**d**); and stimulated with different concentrations of OVA_323–339_-pulsed immature DC or LPS-matured DC (**e**) was measured. Statistical analysis was performed with one-way analysis of variance with Bonferroni's multiple comparison test, ***P<0.001.

In addition to transcriptional factor expression, different subsets of Th cells express a unique set of hallmark cytokines. Previously, we had observed secretion of several Th1-effector cytokines during CD4^+^ Th cell expansion. To examine co-expression of these Th-cytokines, we restimulated antigen-experienced CD4^+^ Th cells that had been expanded in cA-DMEM/F12 with low-dose IL-2 and medium-dose IL-7, with OVA_323-339_-pulsed BMDC in the presence of Brefeldin A (Supplementary Data Schema S4). Co-expression of IL-2, IFN-γ, and TNF-α was observed in majority of the CD4^+^ Th cells. A minority of these cells were also found to co-express IL-17A (Supplementary Figure S5). Bimodal expression of IFN-γ was observed, and cells that expressed lower levels of IFN-γ were also positive for IL-17A expression (Supplementary Figure S5). The expression of these cytokines was comparable between cells expanded for short (10 days) or extended (20 days) periods of time (Figures 4a and b). Granzyme B was expressed by fewer day 10 cells, but dramatically upregulated by day 20 cells (Figures 4a and b). Expression of IL-4 and IL-10 in both day 10 and day 20 cells was found to be much lower compared with other Th1-cytokines (Figures 4a and b). This observation suggests favorable lineage commitment of Th1 cells after clonal expansion with low dose of IL-2 and IL-7.

To evaluate the cytotoxic efficacy of these *in vitro* expanded Th1-like cells, the cells were adoptively transferred into mice that subsequently received OVA_323-339_ peptide-pulsed target cells (Figure 4c). CD4^+^ Th cells expanded with IL-2 and IL-7 induced up to 40% target cells lysis *in vivo* (Figure 4d), and this was not enhanced by blocking IL-4 signaling. Toll-like receptor ligands are able to modulate DC maturation and activation to augment the T-cell response to the target antigen.^26^ We wished to determine whether a different cytolytic capacity of CD4^+^ Th cells would be observed when these cells were stimulated with immature and LPS-mature peptide-pulsed DC. Surprisingly, CD4^+^ Th cells activated with LPS-matured and immature DC showed similar *in vivo* cytolytic capacity (Figure 4e). The CD4^+^ Th cells were also stimulated with DC pulsed with 10 times more OVA_323-339_ and expanded in the same cytokine condition to see whether increase antigen level during T-cell stimulation could enhance their cytolytic activity. Similar cytotoxicity of CD4^+^ T cells were observed, regardless of the amount of peptide antigen used for T-cell stimulation (Figure 4e). These observations indicate activation and expansion with 1 ug ml^−1^ OVA_323–339_-pulsed DC and IL-2 and IL-7 is sufficient to induce cytotoxic CD4^+^ Th cells. Maturation of DC for CD4^+^ T-cell priming is unnecessary to enhance cytolytic function in these cells.

### CD4^+^ Th1-like cells enhance the CD8^+^ CTL-mediated anti-tumor response

**Figure 5:**
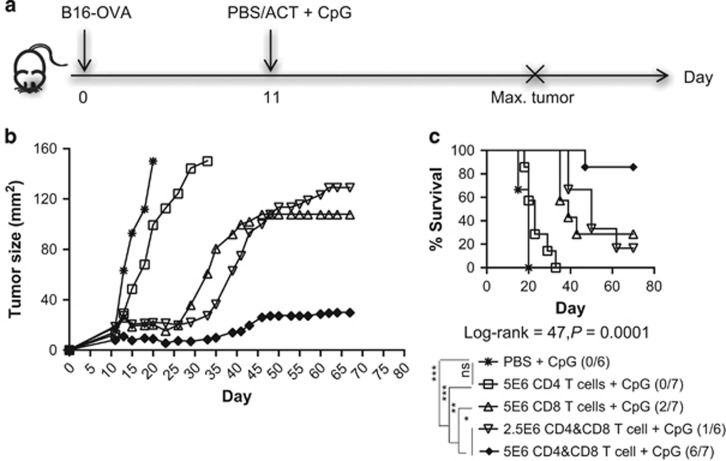
CD4^+^ Th cells enhance anti-tumor rejection induced by CD8^+^ CTL. Naive C57BL/6 mice were (s.c.) injected with 5 × 10^4^ B16-OVA cells on day 0, and randomized into five different groups (*n*=7) when the tumors became palpable. On day 11, the mice were (i.v.) injected with PBS, or 2.5 × 10^6^ or 5 × 10^6^ of each day 20 *in vitro* expanded CD4^+^ OT-II cells and/or CD8^+^ OT-I cells, followed by (s.c.) injection of CpG (20 μg per mouse). (**a**) Tumor growth was monitored and the mice were killed once tumor size reached 150 mm^2^; (**b**) tumor growth curve and (**c**) survival curve. Statistical analysis was performed with Long-rank test for survival, and one-way analysis of variance analysis to compare survival with PBS control. *P<0.05, **P<0.01, ***P<0.001.

To evaluate the efficacy of CD4^+^ Th1-like cells in controlling tumor growth, we expanded CD4^+^ OT-II and CD8^+^ OT-I cells *in vitro* with the method we optimized (Supplementary Data Schema S5), and adoptively transferred them into mice bearing an established B16-OVA melanoma (Figure 5a). Because CpG has been shown to enhance the anti-tumor activity of the adoptively transferred T cells,^27^ it was given locally adjacent to the tumor following T-cell infusion. Despite a similarity in their cytokine profile (Supplementary Figure S6b), CD4 Th1-like cells and CD8^+^ CTL exhibited different abilities to control tumor growth. ACT with 5 × 10^6^ CD4^+^ Th cells alone moderately delayed tumor growth, but no tumor-free survival was observed (Figures 5b and c). We have also performed ACTs using CD4^+^ T cells generated with the same cytokine conditions but in complete DMEM medium (rather than complete advanced-DMEM/F12), and observed no delay of tumor growth in mice treating with these cells (data not shown). Although CD8^+^ CTL could induce more than 90% antigen-specific lysis of their target cells in an *in vivo* cytotoxicity assay (data not shown), they induced less than 30% tumor-free survival as a single-cell therapy (Figure 5c). Similar anti-tumor efficacy could be achieved by giving a combination of 2.5 × 10^6^ CD4^+^ Th cells and CD8^+^ CTL (Figures 5b and c). Doubling the number of these cells in the combination T-cell therapy significantly enhanced tumor regression (Figure 5b), resulting in an 85% tumor-free survival rate (Figure 5c). Intracellular cytokine analysis showed both CD4^+^ Th1-like cells and CD8^+^ CTL were found to express IFN-γ, TNF-α and granzyme B (Supplementary Figure S6), indicating an effector function of these cells. However, IL-2 expression was only detected in CD4^+^ Th1-like cells, but rarely in CD8^+^ CTL (Supplementary Figure S6). This may be the reason why single T-cell therapy with CD8^+^ CTL was less effective in controlling tumor growth, as IL-2 is required to support survival of these cells. However, it is unclear whether these CD4^+^ Th1-like cells primarily function to kill tumors or to assist the effector function of CD8^+^ CTL. Nevertheless, these observations suggest that CD4^+^ Th1-like cells can synergize with CD8^+^ CTL to enhance the immune response to tumors.

## Discussion

In this study, we have developed an *in vitro* cell expansion method to generate Th1-like cells from naive CD4^+^ Th cells, and demonstrated their ability to produce multiple Th1-cytokines, cytotoxicity and enhance CD8^+^ CTL-mediated tumor rejection. Traditionally T-cell expansion in tissue culture has been performed with a high dose of recombinant IL-2. However, IL-2 was found to sensitize CD4^+^ Th cells to activation-induced cell death (AICD) and induced IL-2-dependent survival of these T cells.^28^ Because of this, systemic administration of exogenous IL-2 is required to augment the functional activity of these T cells after adoptive transfer.^29^ In addition to IL-2, IL-7 and IL-15 are also commonly used for expansion of human antigen-specific CD8^+^ T cell in clinical studies of ACT.^30, 31^ To avoid hyper-proliferation induced cell exhaustion, we limited the concentration of cytokines used for T-cell expansion to 10 ng ml^−1^ in this study. IL-7 was found to induce similar CD4^+^ Th cell expansion as IL-2 but resulted in less apoptosis and better survival. This observation is supported by the finding that IL-7 was preferable for *ex vivo* expansion of tumor-specific murine CD4^+^ Th cells compared with IL-2.^32^ IL-15 has been shown to synergize with IL-7 to regulate homeostatic proliferation of memory CD8^+^ T cells but are not required for the proliferation of memory CD4^+^ Th cells.^33^ Similarly, we observed that IL-7 and IL-15 did not synergize to enhance expansion of either naive or antigen-specific CD4^+^ Th cells.

The strength of TCR stimulation can also influence downstream events in T-cell response.^34^ We observed a significant cell death after prolonged TCR restimulation of antigen-experienced cells. It is possible that these cells require only a brief engagement of MHC/peptide complex to commit to proliferation. Therefore we conducted a time course study to examine the relationship between the duration of TCR restimulation and CTLA-4 expression with cell expansion, and found that a short period of 4 h was sufficient for antigen-experienced cells to commit to secondary expansion. This observation is comparable with a previous finding that effector T cells commit to proliferation after a short period of engagement with their target antigen but die following activation if the TCR restimulation was prolonged.^35^

We also examined the effect of different cell media on cell expansion. Compared with cDMEM-5 and cDMEM/LG-5, cA-DMEM/F12-5 was found to induce superior cell expansion. The cA-DMEM/F12-5 medium is specially formulated with the addition of proteins and trace elements to promote cell growth in serum-reduced condition. These ingredients may also serve to promote cell proliferation while maintaining CD4^+^ Th cell differentiation. The cDMEM-5, cDMEM/LG-5 and cA-DMEM/F12-5 are also formulated with different glucose concentrations of 4500, 1000  and 3151 mg l^−1^, respectively. However, the difference in glucose contents in these media might not be sufficient to effect the T-cell expansion. It is also possible that the cA-DMEM/F12-5 promotes better cell expansion and survival through upregulation of CD44 expression, which has been shown to specifically regulate survival in Th1 but not any other subset of Th cells or CD8^+^ CTL.^36^

CD4^+^ T cells with a Th1 phenotype that are capable of producing multiple Th1 cytokines are well known to be important for enhancing CD8^+^ CTL responses during an anti-tumor immunity. A study by Bird *et al.*^37^ showed that more than four cell divisions under the Th-1 polarizing conditions were necessary to drive naive CD4^+^ commitment to Th1 polarization. In addition, another study done by Bajenoff *et al.*^38^ showed that optimal Th1 effector properties could not develop unless the TCR was re-engaged and the cells were exposed continuously to Th1-polarizing cytokines. Generation of Th1-polarized cells in both studies required continuous exposure of these cells under the Th1-polarizing conditions. We have observed that naive CD4^+^ Th cells expanded through one or multiple TCR stimulations without using Th1-polarizing conditions had similar Th1-cytokine profiles and cytolytic capacity, with associated multiple transcription factor expression. The different observations between our study and the studies done by Bird and Bajenoff may be due to the use of different antigen stimuli. Anti-CD3/CD28 was used for TCR stimulation in both previous studies, however, antigen-pulsed DC was used in this study. Transient co-expression of lineage-specific transcription factors in CD4^+^ Th cells has been reported, suggesting interplay between the lineage-specifying transcription factors and lineage plasticity of these CD4 Th1-like cells.^39^ In addition, expression of Foxp3 has been found to be associated with T-cell activation.^40^ Predominant expression of multiple Th1-cytokines indicates these cells were Th1 or Th1-like cells. Indeed, the finding of cytolytic activity in these CD4^+^ Th cell suggests they might be a subset of the newly described T helper CTL. The transcriptional factor of the T helper CTL has yet to be identified. These cells are currently identified by the co-expression of granzyme B, perforin, and surface expression of CD107a.^41, 42^ Although we did not examine the expression of perforin and CD107a in this study, expression of granzyme B by these cells was observed.

Furthermore, we have also shown that CD8^+^ CTL, but not CD4^+^ Th cells, comprise the main subset of T cells responsible for eliminating tumor cells; however, the presence of CD4^+^ Th1-like cells is necessary to enhance complete tumor regression. CD4^+^ Th1 cells have been shown to prolong the survival, the memory response and tumor localization of the antigen-specific CD8^+^ CTL in an animal cancer model.^43, 44^ Co-transferring CD4^+^ and CD8^+^ CTL in ACT may also circumvent the need for systemic IL-2 administration, as CD4^+^ Th1 cells are able to secrete helper cytokine IL-2 to CD8^+^ CTL.^43, 45^ Interestingly, two independent studies have shown that adoptive transfer of 50 000 naive and 1 × 10^6^ Th17 polarized tumor-reactive CD4^+^ Th cells induced complete regression of established murine melanoma.^46, 47^ It is important to recognize that lymphodepletion with prior sublethal irradiation was performed in both studies. Lymphodepletion has been shown to delete immunosuppressive cells, create space and allow homeostatic proliferation of the transferred T cells, leading to a better anti-tumor response.^48^ However, not all cancer patients are susceptible to radiotherapy, due to manifold side-effects, genetic conditions, and the nature of the cancer.^49^ Moreover, the therapeutic effect of the Th17 cells was found to be critically dependent on their ability to convert to Th1 cells and the acquisition of IFN-γ production;^47^ and host lymphodepletion is required to promote Th17 to Th1 conversion.^50^ By contrast, we showed that combining CD4^+^ Th1-like cells and CD8^+^ CTL in ACT without prior sublethal irradiation significantly enhanced anti-tumor rejection, leading to complete cure and long-term survival of the animal.

Here, for the first time, we have explored strategies to generate large-scale *in vitro* expansion of CD4^+^ Th cells in an antigen-specific manner for the purpose of adoptive T-cell therapy. The methodology we optimized allows generation of more than 10 000 fold CD4^+^ Th cell expansion in a short period of time. Functional assays demonstrated that these Th1-like cells were not only directly cytotoxic but also synergized with CD8^+^ CTL to enhance complete tumor rejection. Therefore cancer immunotherapies that include targeting of CD4^+^ Th1-like cells are promising for inducing complete, durable anti-tumor rejection.

## MATERIALS AND Methods

### Mice

Specific-pathogen free 8–12 week old female C57BL/6, OT-I and OT-II mice were obtained from either the Hercus Taieri Research Unit, University of Otago, Dunedin, New Zealand; or from the Biomedical Research Unit, Malaghan Institute of Medical Research, Wellington, New Zealand. All mice used for *in vivo* experiments were age-matched. All experimental protocols were approved by the University of Otago Animal Ethics Committee, Dunedin, New Zealand; or the Animal Ethics Committee, Victoria University, Wellington, New Zealand.

### Cell culture media recipes

Complete IMDM (cIMDM-5): IMDM (Gibco and Invitrogen, Waltham, MA, USA)+1% penicillin/streptomycin (Gibco and Invitrogen)+0.1% 2-mercaptoethanal (Gibco and Invitrogen)+5% fetal calf serum (FCS).

Complete DMEM (cDMEM-5): DMEM+1% P/S+0.1% 2-ME+1% l-glutamine+1% l-arginine+1% l-asparagine+1% folic acid+5% FCS.

Complete DMEM/low glucose (cDMEM/LG-5): DMEM+1% P/S+0.1% 2-ME+1% l-glutamine+1% l-arginine+1% l-asparagine+1% folic acid+5% FCS Complete advance-DMEM/F12 (cA-DMEM/F12-5): advanced-DMEM/F12+ 1% P/S+0.1% 2-ME+1% GlutaMax (Gibco and Invitrogen)+20 mm HEPES solution (Gibco and Invitrogen)+5% FCS.

### Flow cytometric analysis

Unless otherwise stated, all antibodies were purchased from BioLegend, San Diego, CA, USA, and titrated before use. Cell viability staining was performed with Live/Dead fixable dye (Invitrogen) according to the manufacturer's instructions before surface and/or intracellular staining. Antibodies used for flow cytometric analysis recognized the following mouse cell epitopes: CD4 (GK.15); CD44 (1M7); CD62L (MEL-14); CTLA-4 (UC10-4B9); MHC-II (M5/114.15.2); T-bet (4B10), Foxp3 (FJK.16s); GATA-3 (TWAJ, eBioscience, San Diego, CA, USA); IL-2 (JES6-5H5, BD Bioscience); IL-4 (11B11, BD Bioscience); IL-10 (JES5-16E3, BD Bioscience); IL-17A (TC11-18H10, BD Bioscience); IFN-γ (XMG1.2); TNF-α (MP6-XT22, BD Bioscience); and Granzyme B (GB11, eBioscience). All surface staining was performed at 4 °C for 10 min. Intracellular staining was performed with the Foxp3 staining buffer set (Cat# 00-5523-00, eBioscience) according to the manufacturer's instructions. To examine intracellular expression of cytokines, effector/memory CD4^+^ Th cells were stimulated with OVA_323-339_-pulsed DC, 25 ng l^−1^ phorbol 12-myristate 13-acetate (PMA) plus 0.5 μg l^−1^ Ionomycin as a positive control, or unpulsed DC as a negative control, in the presence of 1 × Brefeldin A (BioLegend) in cIMDM-5 (complete IMDM) for 5 h at 37 °C/5% CO2. All flow cytometric analysis was undertaken with either a BD Fortessa or FACS Aria (San Diego, CA, USA) and the data analyzed with FlowJo 9.6 software (Ashland, OR, USA).

### Generation of bone marrow-derived DC

C56BL/6 BMDC were generated as previously described.^51^ Briefly, bone marrow single-cell suspensions were cultured in a six-well-plate (Falcon, St Louis, CA, USA) at 2.5 × 10^6^ per well in cIMDM-5 supplemented with 20 ng l^−1^ recombinant GM-CSF (Biosource) (DC medium). Cultures were fed every 2–3 days by removing 50% of the medium from each well and replenishing with an equal amount of fresh DC medium, and incubated at 37 °C/5% CO_2_ for 6–7 days. For DC maturation, 1 μg l^−1^ lipopolysaccharide (LPS, Sigma Aldrich Co., St Louis, CA, USA) was added to day 6 BMDC cells overnight (O/N).

### Isolation of CD4^+^ OT-II and CD8^+^ OT-I cells, and MHC-I and MHC-II peptides for T-cell stimulation

Antigen-specific CD4^+^ T cells and CD8^+^ T were isolated from OT-II and OT-I splenocytes respectively, through magnetic bead separation. Anti-mouse CD4 (clone L3T4) and CD8 (clone Ly-2) microbeads were obtained from Miltenyi Biotec Ltd (Bergisch Gladbach, Germany), and used according to the manufacturer's instructions. The MHC class I peptide OVA_257–264_ (SIINFEKL) (JPT Peptide, Berlin, Germany) and the MHC class II peptide OVA_323–339_ (ISQAVHAAHAEINEAGR) (Mimotopes Pty Ltd, Clayton, VIC, Australia) of ovalbumin (OVA), were used as the target antigens for CD8^+^ CTL and CD4^+^ Th cells, respectively. Unless otherwise stated, 1 μg l^−1^ peptide concentration was used for both CD4^+^ and CD8^+^ cell stimulation. Non-adherent and loosely adherent cells were harvested, washed, and resuspended at 1 × 10^6^ cells per ml in DC medium; OVA_257-264_ or OVA_323-339_ were added to the cells and incubated for 4 h at 37 °C/5% CO_2._ Free peptides were washed off with dulbecco's phosphate-buffered saline (DPBS), and the DCs were resuspended in cIMDM-5 at 1 × 10^6^ cells per ml for T-cell stimulation.

### Optimization of antigen-specific CD4^+^ Th1 cell expansion *in vitro*

For T-cell clonal expansion, CD4^+^ cells isolated from naive OT-II mice were stimulated with DC-OVA_323-339_ at 1 × 10^6^ cells per ml at a DC to T-cell ratio of 1:10, and expanded in different cytokine conditions and different media at 37 °C/5–10% CO_2_. Unless otherwise stated, cells were expanded for 10 days upon antigenic stimulation. T cells were fed by splitting into new wells every 2–3 days with fresh medium and cytokines were replenished. The cell concentration was maintained at 0.5–1 × 10^6^ cells per ml. Unless otherwise stated, cytokine(s) were added upon TCR stimulation to support the cell expansion. Expansion of naive T cells was referred to as primary (first) expansion; and expansion of effector/memory cells upon restimulation was referred to as secondary (second) expansion. For the positive control, cells were stimulated with a 1:1 ratio of anti-CD3/CD28 beads (Invitrogen); for the negative control, cells were stimulated with unpulsed DC. All controls were maintained with 1 ng ml^−1^ IL-2 and 5 ng ml^−1^ IL-7, and fed as cells expanded in different culture conditions. The number of viable cells was counted at the end of each cell expansion using a hemocytometer and trypan blue was used to exclude dead cells. Fold of cell expansion was calculated as: final number of viable cells per number of viable cells on the day of antigen stimulation.

### Titration of IL-2, IL-7 and IL-15

Naive CD4^+^ OT-II cells were stimulated with DC-OVA_323-339_ and expanded for 20 days at 37 °C/5% CO_2_ in cDMEM-5 in a 24-well plate (1 ml per well), during which the T cells were restimulated on day 10 with DC-OVA_323-339_ for secondary expansion. Medium only, 1 , 5 or 10 ng ml^−1^ of recombinant IL-2 (BioLegend) and/or IL-7 (Gibco, Invitrogen) and/or IL-15 (BioLegend) were added for the entire cell expansion upon TCR stimulation.

### Time course restimulation of antigen-experienced T cells

Naive CD4^+^ OT-II cells were stimulated with DC-OVA_323-339_ and expanded with 1 ng ml^−1^ IL-2 and 5 ng ml^−1^ IL-7 in cDMEM-5 for 10 days; and restimulated with either 0.2  or 1 μg ml^−1^ OVA_323–339_-pused DC, for 4, 8, 24 or 72 h. Non-adhesive T cells were gently pipetted up and down then transferred into new wells for secondary expansion with 1 ng ml^−1^ IL-2+5 ng ml^−1^ IL-7 until day 20. Surface expression of CTLA-4 on CD4^+^ cells that restimulated for different period of time point was measured by flow cytometric analysis.

### Expansion of CD4^+^ Th cells in different media

Naive CD4^+^ OT-II cells (1 × 10^6^ cells per well in a 12-well plate) were stimulated with DC-OVA_323-339_ and expanded in cDMEM-5 initially. On days 10 and 20, antigen-experienced T cells were stimulated for 4 h with DC-OVA_323-339_ and in cDMEM-5, cDMEM/LG-5 or cA-DMEM/F12-5. IL-2 (1 ng ml^−1^) was added only upon TCR stimulation and IL-7 (5 ng ml^−1^) for the entire cell expansion. Cell supernatant was collected 3 days after TCR stimulation to examine cytokine secretion. Expression of CD44 and CD62L was measured on day 10 and day 20 cells by flow cytometric analysis.

### Polarization of CD4^+^ Th1 cell expansion

Naive CD4^+^ OT-II were stimulated with DC-OVA_323-339_, and expanded with IL-2 and IL-7 in A-DMEM/F12 for 20 days as previously described in section 4.6.3. Two different protocols were used to induce polarized CD4^+^ Th cell expansion. To induce polarized Th1 cell differentiation, either 1 ng ml^−1^ IL-12 (Biolegend) or 1 μg ml^−1^ of anti-IL-4 (BioLegend) was added in addition to IL-2 and IL-7.

### Measurement of cytokine production by CD4^+^ Th cells during clonal expansion

To measure the amount of cytokines secreted during CD4^+^ Th cell expansion, culture supernatant was collected 3 days after TCR stimulation and the concentrations of IL-2 (detection and capture Ab Clone: JES6-5H4, BD Biosciences), IL-12 (detection and capture Ab Clone: C15.6, BD Biosciences), IL-17A (ELISA MAX standard kit, BioLegend), TNF-α (detection and capture Ab Clone: MP6-XT22, BioLegend) and IFN-γ (detection and capture Ab Clone: XMG1.2, BD Biosciences) were quantified by ELISA according to the manufacturer's instructions. IL-4, IL-10 and TGF-β were measured with Milliplex kits following the manufacturer's instructions.

### Expansion of CD4^+^ Th cells and CD8^+^ CTL for functional assays

Unless otherwise stated, naive CD4^+^ OT-II cells and CD8^+^ OT-I cells were stimulated with immature DC-OVA_323-339_ and LPS-matured SIINFEKL-pulsed BMDC (LPS/DC-S) at a DC:T-cell ratio of 1:10, respectively; and expanded in cA-DMEM/F12-5 for either 10 or 20 days. For 20 days' cell expansion, the T cells were restimulated with peptide-pulsed DC for 4 h as described in section 4.6.2. IL-7 was added at 5 ng ml^−1^ to both cell types throughout the entire period of cell expansion; and 1 ng ml^−1^ IL-2 was added to CD4^+^ Th cells only upon TCR stimulation.

### Cytotoxicity assays

Naive CD4^+^ OT-II cells were stimulated with 1 or 10 μg ml^−1^ OVA_323-339_ pulsed immature or LPS-matured BMDC, and expanded with IL-2 (1 ng ml^−1^) and IL-7 (5 ng ml^−1^) +/− anti-IL-4 (1 μg ml^−1^) in A-DMEM/F12-5 for 20 days. To test the *in vivo* cytotoxicity of these CD4^+^ Th cells, naive C57BL/6-recipient mice (*n*=3–4) were intravenously (i.v.) injected with either PBS, or 2 × 10^6^ *in vitro* expanded CD4^+^ Th cells on day 0. Splenocytes from naive C57BL/6 mice were either pulsed with 5 μg ml^−1^ OVA_323-339_ (target), or left unpulsed (control) at 5 × 10^6^ cells per ml in cIMEM-5 for 3 h at 37 °C/5% CO_2_. Target and control cells were incubated with 2 μm or 0.2 μm CFSE respectively, at room temperature for 8 min. One volume FCS was added to quench the reaction and the cells were washed three times with 20 volume DPBS then mixed at 1:1 ratio at 50 × 10^6^ cells per ml; 200 μl of this cells mixture was injected i.v. into mice 24 h post-effector cell infusion. The mice were killed 40 h post-target cell transfer, and spleens were resected. Splenocytes were stained with Live/Dead fixable dye, followed by MHC-II, and then analyzed by flow cytometry. Percent lysis was calculated using the formula: Ratio=# Control/# peptide-pulsed, % specific lysis=1–(ratio_absence of E_/ratio_presence of E_) × 100.

### Adoptive T-cell therapies (ACT) in B16-OVA model

For ACT, naive C57BL/6 mice were subcutaneously inoculated with 5 × 10^4^ B16-OVA cells and randomized into different groups (*n*=7) when the tumors become palpable on day 7. On day 11, the mice were intravenously injected with DPBS, 5 × 10^6^ day 20 CD4^+^ or 2.5 × 10^6^ of each *in vitro* expanded CD4^+^ and/or CD8^+^ cells. The mice also received subcutaneously CpG (20 μg per mouse in 50 μl DPBS) adjacent to the tumor after the T-cell infusion. Tumor growth was monitored, and the mice were killed when the tumors reached 150 mm^2^ size.

### Statistical analysis

Statistical analysis was performed with Graph Pad Prism 6 (La Jolla, CA, USA). The particular type of statistical analysis is listed on each relevant figure legend. *P*-value<0.5 was considered to be statistically significant.
