# Supplementary material for: Conditions for the generation of cytotoxic CD4+ Th cells that enhance CD8+ CTL-mediated tumor regression
Source: Clin Transl Immunology. 2016 Aug 12;5(8):e95–. doi: 10.1038/cti.2016.46 (PMC5007627; doi:10.1038/cti.2016.46)
Supplement: Supplementary Information [file cti201646x1.docx]

# Supplementary data

Schema 1: Titration of IL-2, IL-7 and IL-15 for CD4 T cell expansion.

Schema 2: Cell expansion in different media.

Schema 3: polarization of CD4 Th cell expansion

Schema 4: CD4 T cell expansion for cytokine assay and *in vivo* cytotoxic assay

Schema 5: T cell expansion for ACT

Figure S1: Titration of IL-2, IL-7 and IL-15 for CD4 Th cell expansion

Naïve CD4^+^ OT-II cells were stimulated with DC-OVA_323-339_ and expanded in IL-2, and/or IL-7, and/or IL-15 in cDMEM for 20 days. TCR restimulation with DC-OVA_323-339_ for 4 hr was performed on day 10; (a) primary expansion and (b) secondary expansion. Alternatively, TCR restimulation was performed on day 14 after 4 days resting; (c) primary cell expansion, (d) secondary cell expansion, and e) cell survival after resting for 4 days. The results are the mean ± SEM of 3 independent experiments. Statistical analysis was performed with two-way ANOVA with Bonferroni’s multiple comparison test to compare against cells expanded without cytokine supplementation, * = P<0.05, ** = P<0.01, *** = P<0.001, **** = P<0.0001.

Medium

1

5

10

Medium

1

5

10

IL-2 (ng/ml)

IL-7 (ng/ml)

Figure S2: IL-7R expression by day 20 CD4 T cells

Figure S3: Transcriptional factor and cytokine expression by CD4 Th cells expanded with the addition of exogenous IL-12

CD4^+^ cells isolated from naïve OT-II mice were co-cultured with DC-OVA_323-339_ and expanded with 3 different cytokine conditions in cA-DMEM/F12 for 20 days, during which the cells were restimulated on day 10. Cell supernatant was collected 3 days after antigenic stimulation. The presence of IL-2 and IL-12 in culture supernatant after 72 hr of CD4 Th cell and DC-OCA_323-339_ co-culture was measured by ELISA; and IL-4 and IL-10 was measured with Bioplex kit (Milipore); dash line indicates the level of IL-2 and IL-12 added upon antigen stimulation. Expression of GATA-3 and Foxp-3 by cells at the end of 10 and 20 days expansion was measured by flow cytometry (b). The results are combined from three independent experiments, showing mean ± SEM. Statistical analysis was performed with two-way ANOVA with Bonferroni’s multiple comparisons test, * = P<0.05, ** = P <0.01.

**Figure S4: Transcriptional factor and cytokine expression by CD4 Th cells expanded with the addition of anti-IL-4 mAb**

Naïve CD4 Th cells were stimulated with DC-OVA_323-339_ and expanded with IL-2 and IL-7 +/- anti-IL-4 mAb (1 μg/ml) in cA-DMEM/F12 for 20 days, during which the cells were restimulated on day 10. Expression of T-bet and Foxp-3 was measured by flow cytometric analysis. The results are combined from three independent experiments, showing mean ± SEM. Statistical analysis was performed with one-way ANOVA with Bonferroni’s multiple comparison test, no statistical significant was observed.

**Figure S5: Co- expression of Th cell cytokines by *in vitro* expanded CD4 Th cells.**

Day 10 *in* *vitro* expanded CD4 Th cells were re-stimulated with DC-OVA_323-339_ or PMA/Ionomycin in the presence of Brefeldin A, or left untreated for 5 hr at 37˚C/5% CO_2_. Cells were harvested and stained with Live/Dead fixable stain, followed by intracellular staining with mAb against mouse IL-2, IL-4, IL-10, IL-17A, Granzyme B, IFN-γ and TNF-α. Cells were then analysed with flow cytometry and the data was analysed with FlowJo software. The FACS data is a representative of cells re-stimulated with DC-OVA_323-339_ from 4 independent experiments with similar results; a) gating strategy and b) co-expression of cytokines by CD4 T cells.

**Figure S6: phenotypic analysis and cytokine expression of day 20 CD4 and CD8 CTL used for ACT.**

CD4 Th1-like cells and CD8 CTL were expanded in c-A-DMEM/F12-5 for 20 days with 4 hr restimulatuion, and used for ACT in B16-OVA-bearing mice. The phenotype and cytokine profile of these cells were examined with flow cytometric analysis.
